# Patients with treated autoimmune hepatitis and persistent suppression of plasmacytoid dendritic cells: A different point of view

**DOI:** 10.1177/20587384211068667

**Published:** 2022-04-11

**Authors:** Irene P dos Santos, Mayra T de Assunção, Renan M Mauch, Natascha Silva Sandy, Marcos Tadeu Nolasco da Silva, Maria Angela Bellomo-Brandão, Adriana Gut Lopes Riccetto

**Affiliations:** 1 Center for Hematology and Hemotherapy, 28132 University of Campinas, Campinas, Brazil; 2 Pediatric Gastroenterology Clinic, Hospital de Clínicas (University of Campinas Teaching Hospital), 28132 University of Campinas, Campinas, Brazil; 3 Center for Investigation in Pediatrics, School of Medical Sciences, 28132 University of Campinas, Campinas, Brazil; 4 Division of Gastroenterology, Hepatology and Nutrition, Department of Pediatrics, 7979 Hospital for Sick Children, Toronto, ON, Canada; 5 Department of Pediatrics, School of Medical Sciences, University of Campinas, Campinas, Brazil

**Keywords:** plasmacytoid dendritic cells, autoimmune hepatitis, flow cytometry

## Abstract

**Objectives:** Plasmacytoid dendritic cells (pDCs) have been shown to have a role in autoimmune diseases, but their role in Autoimmune Hepatitis (AIH) is not completely clear. In the present study, we assessed the frequency of pDCs in peripheral blood of AIH patients under long-term standard immunosuppressive therapy. **Methods:** This cross-sectional analysis enrolled 27 AIH patients and 27 healthy controls. We analyzed and compared their proportion of pDCs, CD4^+^, CD8^+^, γδ T cells, CD25^+^ regulatory T (Treg) cells, FoxP3^+^, Foxp3^+^CD39^+^ Treg cells, total B (CD19^+^) cells, and plasma cells (CD38^+^) in peripheral blood using flow cytometry immunophenotyping. **Results:** AIH patients had a lower percentage of pDCs (median frequencies of 0.2% vs. 0.4%; *p* = .002) and higher expression of CD8 T cells (32.5% vs 28.6%; *p* = 0.008) in peripheral blood, when compared to healthy controls. We did not find statistically significant differences between the groups regarding the other cell subtypes.**Conclusion:** Our data suggest a persistent suppression of pDCs in AIH patients, along with increased CD8 T cell activity, years after AIH diagnosis and despite of good clinical response to treatment, thus pointing to a role of pDCs in the AIH pathogenesis.

## Introduction

Autoimmune hepatitis (AIH) can affect pediatric and adult individuals in a progressive manner and is marked by clinical, biochemical and histologic findings, and autoantibody production. The current knowledge on AIH pathogenesis supports the role of environmental triggers, uncontrolled activation of T and B lymphocytes, poor inflammation control by T cells, and abnormal activity of macrophages and natural killer cells, leading to a necroinflammatory and fibrotic liver disease.^1–3^

Recent studies have focused on the role of dendritic cells (DCs) in the pathogenesis of several immune-mediated conditions, including AIH.^4–10^ DCs are Antigen-Presenting Cells (APCs) and are well known to be crucial for linking the innate and adaptive immune responses and balancing tolerance to self-antigens in auto‐inflammatory responses.^11^ Multiple DCs subsets have been recognized in mammalians. Flow cytometry and mass cytometry analyses of mice, macaque, and human tissues have identified lineage-imprinted markers that subdivide DCs into type 1 and 2 conventional DCs (cDC1s and cDC2s, respectively), and plasmacytoid DCs (pDCs).^12^

Conventional DCs are specialized APCs that migrate to the T cell zones of draining lymph nodes in a CCR7-dependent manner and play a surveillance role in the tissues. The pDCs comprise a different DC subset that express the type-C lectin Blood Dendritic Cell Antigen 2 (BDCA2).^13^ They have distinct functions and migratory pathways, and varied responses to the same stimuli. When challenged with an antigen via toll-like receptor (TLR)7 or 9, they produce type I interferon (IFN)—IFN-alpha (α) or -beta (β)—through the mTOR signaling pathway.^12,14,15^ It has been suggested that this continuous stimulation of the immune system by IFN production is involved in the development of autoimmunity.^8,16,17^ However, the role of pDCs in the AIH pathogenesis is not completely clear.

In this context, the aim of the present study was to evaluate the frequency of pDCs in peripheral blood of Brazilian patients diagnosed with AIH who were under long-term standard immunosuppressive therapy and to compare their pDC frequency with healthy controls. We also assessed the frequency of CD4^+^, CD8^+^, γδ T cells, regulatory T (Treg) cells, CD25^+^, CD25+FoxP3+, CD25+Foxp3+CD39^+^ Treg cells, and B cells in their peripheral blood using a flow cytometry-based assay.

## Materials and methods

### Study participants and design

In this cross-sectional analysis, we included a convenience sample of children and adults followed at the Pediatric Hepatology Reference Center, located at the University of Campinas Teaching Hospital (HC Unicamp), in Campinas, Brazil. Ethical approval for this study was obtained from the University of Campinas Research Ethics Committee — Certificate for Ethical Appreciation 84944018.7.000.5404; approval number 3.566.946, 9 November 2019. Written informed consent was obtained from all subjects and/or their legal guardians. Patients were enrolled if: (1) they had been diagnosed with AIH during childhood, following well-established and internationally recognized diagnostic criteria^18,19^; (2) if they had been under standard immunosuppressive therapy for this condition for at least 6 months.^19^ We did not include patients who had undergone a liver transplant, patients who had been diagnosed with other autoimmune diseases, and/or overlapped with sclerosing cholangitis.

The control group consisted of healthy individuals aged 18–25 years old. Upon recruitment, all individuals reported having no chronic or autoimmune diseases, no chronic use of any medication, no history of smoking, alcohol, or drug abuse. They also underwent a careful assessment of their health status by means of structured anamnesis, performed by a gastroenterologist.

### Flow cytometry immunophenotyping

Peripheral blood samples (10 mL) from patients and healthy individuals were collected in tubes with ethylenediaminetetraacetic acid. The tubes were incubated for 20 min at room temperature (RT) in the dark with monoclonal antibodies targeting the surface markers CD45 (PerCP, BD), CD3 (V500, BD), CD4 (V450, BD), CD8 (BB515, BD), CD25 (BB515, BD), FoxP3 (Alexa Fluor-647, BD), CD39 (PE, BD), TCR-αβ (APC, BD), CD19 (APC-Cy7, BD), CD38 (PE, BD) and BDCA2 (APC, eBioscience). After incubation, red blood cells were lysed with 2 mL of erythrocyte lysis buffer (BD FACS™ Lysing Solution, BD Biosciences) for 10 min at RT in the dark. The samples were then spun at 1500 rpm for 5 min, washed twice with 2 mL of phosphate-buffered saline (PBS) solution (each washing step was followed by centrifugation at 1500 r/min for 5 min). Cells labeled with fluorophores conjugated to CD4, and CD39 were fixed and resuspended in Forkhead box P3 transcription factor (FoxP3) permeabilization buffer, according to the manufacturer’s instructions, and labeled with anti-FoxP3 fluorophore-conjugated antibodies for 30 min at RT in the dark.

After the final labeling stage, the cells were washed with 2 mL of PBS, resuspended in 400 μL of PBS and we proceeded to cell acquisition in a six-color BD FACSCanto II cytometer (BD Biosciences). A total of 50,000 events were counted and analyzed using the FACSDiva software (BD Biosciences, USA), in which appropriate gating strategies were used for the subpopulations of interest. First, the events were plotted according to forward and side light scatters (FSC × SSC). All cell populations were selected for their expression of the leukocyte pan-marker CD45 (CD45^+^). Within the CD45^+^ gate, we gated the single cells. From the single cell gate, T (CD3^+^) cells were selected and, within the CD3^+^ gate, we determined the percentages of helper (CD4^+^) and cytotoxic (CD8^+^) T cells (TCR-αβ T cells). Non-TCR-αβ T cells were regarded as TCR-γδ T cells. Treg cells were selected from the CD4^+^ gate. Among the Treg cells, we gated those expressing high amounts of CD25 (CD4^+^CD25^hi^) and those expressing Foxp3 (CD4^+^Foxp3^+^). Within the CD4^+^Foxp3^+^ cells, we gated cells expressing CD39 (CD39^+^). B (CD19^+^) lymphocytes were gated within the CD3^-^ cells. Within the B cell population, we gated the plasma cells (CD38^+^). Finally, pDCs (CD45^+^BDCA2^+^) were gated within cells that were negative for both CD3 and CD19.

### Liver function tests

To assess patients’ hepatic function, we measured their serum levels of total gamma globulins, total immunoglobulin (Ig)A, IgM, IgG, albumin, total bilirubin, direct bilirubin, gamma glutamyl transferase (GGT), alkaline phosphatase (ALP), aspartate aminotransferase (AST), alanine aminotransferase (ALT), and the international normalized ratio (INR).

### Assessment of the response to treatment

The definition of good response to treatment was adapted from Alvarez et al.^18^: symptomatic improvement associated to normalized transaminase levels, partial clinical improvement, reduction by 50% or more in the levels of biochemical markers of hepatic function during the first month of treatment, with decreasing AST and ALT until reaching the desired level—twice as much as the upper reference levels—within a 6-month period after reducing the maintenance therapy. Immunosuppressant therapy included a combination of prednisone (1.5 mg/kg/day, maximum of 60 mg/day) and azathioprine (1.0–1.5 mg/kg/day). The prednisone dose was progressively reduced until reaching the maintenance dose (2.5–5.0 mg/day).

### Statistical analysis

The results of flow cytometric immunophenotyping analysis were expressed as percentages of each cell population in relation to the total proportion of cells in their corresponding gate. The distribution of each cell subtype was summarized with descriptive analysis, using median percentages and interquartile ranges. The data were plotted and analyzed using GraphPad Prism version 5.0 (GraphPad Software Inc, San Diego, CA). Comparisons between AIH patients and controls were done using the two-tailed Mann–Whitney test in SPSS® for Windows, version 17.0.0. (SPSS Inc., USA). A *p*-value ≤ 0.05 indicated statistical significance.

## Results

### Patients’ characteristics

We enrolled 27 AIH patients, who were evaluated from January 2016 to January 2018. Most patients were female, with a median age at AIH diagnosis of 11.3 years and a median age of 16.0 years at the time of enrollment in the study. Nineteen (70.4%) patients had AIH type 1. All patients received standard first-line therapy for AIH, that is, prednisone and azathioprine. The median duration of follow-up at the time of enrollment was 6.5 years, all patients were asymptomatic, and most patients had increased serum levels of GGT, ALP, AST and ALT, total IgA, IgM, IgG, total and direct bilirubin, and INR at the moment of AIH diagnosis (Table 1). The control group consisted of 28 individuals, most of whom were female, with a median age of 22.3 years.

**Table 1.** Demographic characteristics, autoimmune hepatitis phenotype and concentration of different serum biomarkers in patients with autoimmune hepatitis under immunosuppressant therapy seen at the HC Unicamp pediatric hepatology reference center. The data concern the moment of autoimmune hepatitis diagnosis.

Characteristics	*N* (%) of patients or median value (min-max; interquartile Range)
Clinical and demographic data	
Gender (female, male)	25/27 (92.6%), 2/27 (7.4%)
Age at diagnosis (years)	11.3 (1.5–15.3; 5.4)
Age at enrollment in the study (years)	16.0 (1.5–35.9)
Autoimmune Hepatitis type I	19/27 (70.4%)
Concentration of serum biomarkers	
Gamma globulin (g/dL)	2.7 (0.9–4.3; 2.7)
Immunoglobulin A (mg/dL)	173.0 (99.8–713.0; 145.0)
Immunoglobulin M (mg/dL)	164.5 (81.9–366.0; 99.2)
Immunoglobulin G (mg/dL)	1570.0 (752.0–8070.0; 880)
Albumin (g/dL)	3.8 (2.6–4.7; 1.0)
Total bilirubin (mg/dL)	1.4 (0.3–8.3; 2.9)
Direct bilirubin (mg/dL)	1.6 (0.2–9.0; 2.6)
International normalized ratio (INR)	1.3 (1.0–1.6; 0.4)
Gamma glutamyl transferase (GGT, U/L)	120.0 (28.0–283.0; 99.5)
Alkaline phosphatase (ALP, U/L)	316.5 (56.0–1112.0; 270.2)
Aspartate aminotransferase (AST, U/L)	170.0 (30.0–970.0; 418.8)
Alanine aminotransferase (ALT, U/L)	188.5 (16.0–1904.0; 731.3)

### Frequency of plasmacytoid dendritic cells and other cell types in peripheral blood

**Figure 1.**
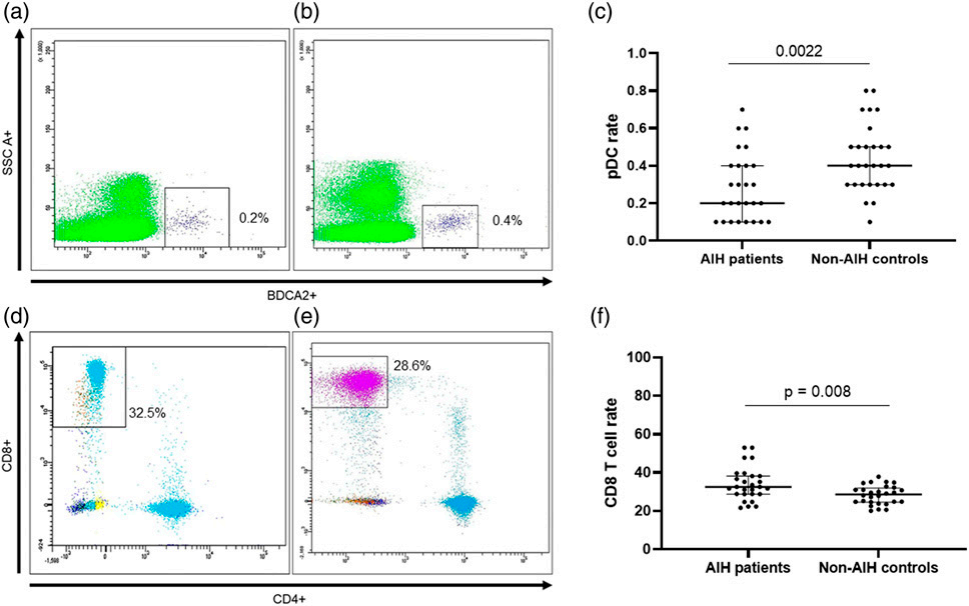
Flow cytometry plots showing the median rate of pDC in (a) patients with AIH under immunosuppressant therapy and (b) healthy controls, and comparison between both groups in a dot plot graphic (c). Flow cytometry plot showing the median CD8^+^ T cells in (d) patients with AIH under immunosuppressant therapy and (e) healthy controls, and comparison between both groups in a dot plot graphic (f). The middle bar in the dot plots indicates the median, the lower and upper bars indicate the first and third quartiles, respectively. AIH: Autoimmune Hepatitis

**Figure 2.**
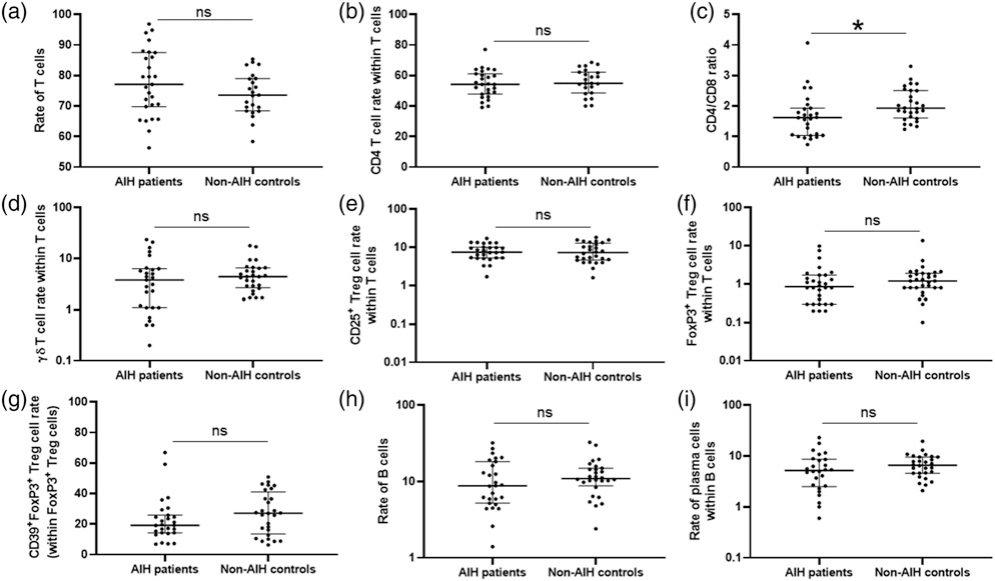
Dot plot graphics showing the (a) rate of T (CD3^+^) cells, (b) rate of helper (CD4^+^) T cells between all T cells, (c) ratio between CD4^+^ and CD8^+^ T cells within T cells, (d) rate of γδ T cells within T cells, (e) rate of regulatory T (Treg) cells expressing (CD25^+^) within T cells, (f) rate of Treg cells expressing FoxP3 (CD3^+^CD4^+^FoxP3^+^) within T cells, (g) rate of Treg cells expressing FoxP3 and CD39 (CD3^+^CD4^+^FoxP3^+^CD39^+^), (h) rate of B (CD19^+^) cells, and (i) rate of plasma cells (CD19^+^CD38^+^) within B cells. The middle bars indicate the medians; the lower and upper bars indicate the first and third quartiles, respectively. (ns) not significant; *p ≤ 0.05.

Autoimmune hepatitis patients had a significantly lower rate of pDCs (CD45^+^BDCA2^+^ cells) than healthy controls (0.2% vs 0.4%). The median rate of CD8 T cells was significantly higher in AIH patients than in controls (32.5% vs 28.6%), and the ratio between CD4 and CD8 T cells was significantly lower in AIH patients than in controls (1.6 vs 1.9) (Table 2, Figure 1). No statistically significant differences were seen between the groups regarding the other cell subtypes (Table 2, Figure 2).

**Table 2.** Rates of different cell subtypes in patients with autoimmune hepatitis (AIH) under immunosuppressant therapy seen at the HC Unicamp Pediatric Hepatology Reference Center and non-AIH controls.

Cell subtype	Median rate (minimum-maximum; IQR)	
	Autoimmune hepatitis patients	Controls
Plasmacytoid Dendritic Cells (pDC)	0.2 (0.1–0.7; 0.3)	0.4 (0.1–0.8; 0.2)
T cells (all) Out of which	77.1 (56.3–96.9; 17.7)	73.6 (58.4–89.5; 11.1)
Helper (CD4) T cells	54.2 (39.1–77.1; 13.2)	57.4 (40.0–68.6; 11.4)
Cytotoxic (CD8) T cells	32.5 (22.4–53.0; 9.3)	28.6 (20.1–37.9; 7.3)
CD4/CD8 T cell ratio	1.6 (0.7–4.1; 0.9)	1.9 (1.2–3.3; 0.9)
γδ T cells	3.8 (0.2–23.5; 5.2)	4.4 (1.6–17.8; 3.9)
Treg cells expressing CD25^a^	7.5 (1.7*–*17.2; 6.7)	7.3 (1.6*–*18.3; 8.3)
Treg cells expressing FoxP3^a^	0.8 (0.2–4.8; 0.9)	1.2 (0.1–2.2; 1.0)
Out of which		
Cells expressing CD39	19.1 (6.9–66.9; 12.0)	27.2 (6.4–50.9; 27.5)
B cells (all)	8.8 (1.4–32.0; 13.0)	11.0 (2.4–32.7; 6.1)
Out of which		
Plasma cells	5.2 (0.6–23.1; 6.2)	6.6 (2.1–19.5; 5.0)

FoxP3: Forkhead Box P3 transcription factor.

^a^Within the CD4+ population

### Response to treatment

Out of 27 patients, 23 showed a good response to treatment. One patient had a good initial response, but their transaminase levels increased again due to inadequate adherence to treatment. Two patients had a partial response to treatment and one patient initiated the treatment during advanced hepatic cirrhosis stage and we were therefore unable to assess a decrease in the transaminase levels.

## Discussion

To the best of our knowledge, this is the first study to evaluate the suppression of pDCs years after AIH diagnosis and established treatment. Our findings are in line with recent translational and clinical research articles suggestive of a protective role of pDCs against immune-mediated liver injury^10^ and previous findings of persistent immune activity signals in patients with AIH even after a good response to standard therapy.^20^

To date, the roles of DCs, pDCs and cDCs in AIH are still not fully elucidated. Recently, there is a growing interest in assessing the role of DCs in AIH and the possibility of targeting these cells with potential therapeutic applications in the management of autoimmune liver diseases.^4–6,21^ Initially, the role of cDCs was described in a mouse model of Concanavalin A (ConA)-induced hepatitis. Depletion of cDCs significantly reduced the severity of liver injury, suggesting a detrimental role of cDCs in the T cell-dependent liver injury.^4^ Conversely, pDCs demonstrated an apparently protective role against immune-mediated acute liver injury in both humans and mice in other studies. This so-called “unexpected immunosuppressive role” was shown to be mediated by IL-35, as heterodimeric components of IL-35 (Il12a and Ebi3) were highly expressed in pDCs and CD4^+^CD25^+^ Treg cells.^10^ A recent review discussed the DC-T cell crosstalk in liver fibrogenesis and hepatocarcinogenesis. As the liver is continuously exposed to harmful antigens, tolerogenic signals are emitted in the hepatic tissue to induce a low T cell response and avoid damage to self-structures, which is characterized by CD4 and CD8 T cell anergy. A potential protective mechanism of pDCs may be driven by induction of a tolerogenic phenotype in APCs by the Cytotoxic T-Lymphocyte-Associated Protein 4 (CTLA-4), through increased production of indoleamine 2,3-deoxygenase, allowing these cells to induce the conversion of naïve T cells into Treg cells.^22^ Like we have found in AIH, a reduced frequency of circulating pDCs was described in patients with acute and chronic viral hepatitis B and C^23,24^

We also corroborate the findings by Koda et al. of reduced pDC frequency both in the liver tissue and in peripheral blood of patients with acute AIH.^10^ However, until now, this had not been addressed in the context of long-term treatment. We now demonstrate that circulating pDCs are still reduced several years after treatment in AIH patients, although they have persistent activation of immune cells, raising the question if the pDC suppression can play a central role in the pathophysiology of the chronic downregulation of immunoregulatory mechanisms seen in AIH, as DCs regulate proliferation and cytokine responses of CD4 and CD8 T cells, via IFN-I production.^25^

Experimental studies with pDC-depleted mice challenged with viruses known to activate pDCs have found that pDC ablation impacts early interferon responses and influences the accumulation of virus-specific natural killer (NK) or CD8 T cells in a virus-dependent manner. A reduction in the early IFN-I production in response to cytomegalovirus infection led to increased viral burden, facilitating the expansion of NK cells, while during vesicular stomatitis virus infection, and enhanced early viral replication led to impaired survival and accumulation of virus-specific CD8 T cells.^26^ On the other hand, in mice models of acute nonviral inflammation, pDCs were shown to undergo apoptosis induced by the activity of CD8 T cells through the perforin pathway, suggesting that cytotoxic T cells are the main players in the control of pDC survival during acute inflammation caused by massive T cell activation.^27^ This is possibly also the case in our cohort of AIH patients, as we found CD8 expansion despite standard therapy, a persistent immune alteration that has been previously reported in patients with type 1 AIH under standard immunosuppressive treatment.^28^

It is worth highlighting that the patients assessed in our study had higher rates of peripheral CD8 T cells even with normal rates of peripheral Treg cells. The latter are commonly impaired, both functionally and numerically, in AIH, although their rates have been shown to be higher in the hepatic tissue. Still, several reports suggest that peripheral Treg cells of AIH patients are impaired in their ability to regulate the proliferation and activation of conventional T cells, including an impaired ability to suppress interferon-gamma (IFN-γ) production and proliferation of CD8 T cells.^29^ Even Treg cells present in the inflamed hepatic tissue, which apparently have normal activity, may be involved in the AIH pathogenesis due to characteristics of the hepatic milieu, such as deficiency of IL-2, an essential cytokine for Treg function.^30^ In the context of treated AIH, normal peripheral Treg cell rates could be a consequence of the immunosuppressive therapy, which, among other mechanisms, decreases the activity of self-reactive T and B cells and boost the activity of Treg cells.^31^

Our study has a number of limitations, including its cross-sectional design and a small sample size. A longitudinal study would have allowed us to investigate and compare changes in subsets of immune cells in different disease and treatment stages. Our definition of disease remission was based on histology, so a variable degree of persistent liver tissue inflammation could not be ruled out or assessed to allow comparison with peripheral findings. In addition, we did not perform functional assays, like cytokine, granzyme, and perforin analyses, which could have provided us with important insights about the activity of CD4 and CD8 T cells. Finally, a previous study has shown that Systemic Lupus Erythematosus (SLE) and AIH can reach 14% of similarity in their pathogenesis,^30^ an overlap that has been described in pediatric patients.^32^ It is speculated that DNA and RNA release by apoptotic cells in patients with SLE are potent stimuli to pDCs, via TLR-7 and -9 activation and high IFN-I production. Patients with SLE were shown to have increased peripheral IFN-I levels and activated pDCs in the spleen,^33^ raising the question whether increased pDCs in peripheral blood samples of our patients could be caused by non-identified SLE, which should be investigated, although the association between pDCs, AIH and SLE remains unclear. Further investigation should be focused on longitudinal studies, analyses of liver biopsies, cytokine production, and studies including AIH patients without a good response to AIH treatment and/or under second and third-line therapy. These analyses may also help to clarify how other immune abnormalities in AIH influence the pDC suppression and if pDC suppression is driven by immunosuppressive therapy. Should the latter be the case, it will also need to be investigated in other hepatic disease models.

## Conclusion

Our results suggest a lower circulation of plasmacytoid dendritic cells in patients with AIH, when compared to healthy controls. These patients also have a higher number of circulating CD8 T cells. We corroborate previous reports showing a rreduction of pDCs in peripheral blood and suggesting a protective role of pDCs in the AIH pathogenesis. Finally, we present new findings that suggest a persistent suppression of pDCs several years after AIH diagnosis and good clinical and biochemical response to standard immunosuppressive therapy.

## Supplemental Material

sj-png-1-iji-10.1177_20587384211068667 – Supplemental Material for Patients with treated autoimmune hepatitis and persistent suppression of plasmacytoid dendritic cells: A different point of view Click here for additional data file. Supplemental Material, sj-png-1-iji-10.1177_20587384211068667 for Patients with treated autoimmune hepatitis and persistent suppression of plasmacytoid dendritic cells: A different point of view by Irene P dos Santos, Mayra T de Assunção, Renan M Mauch, Natascha Silva Sandy, Marcos Tadeu Nolasco da Silva, Maria Angela Bellomo-Brandão and Adriana Gut Lopes Riccetto in International Journal of Immunopathology and Pharmacology
