# Supplementary figures and images for: Patients with treated autoimmune hepatitis and persistent suppression of plasmacytoid dendritic cells: A different point of view
Source: Int J Immunopathol Pharmacol. 2022 Apr 11;36:20587384211068667. doi: 10.1177/20587384211068667 (PMC9006358; doi:10.1177/20587384211068667)

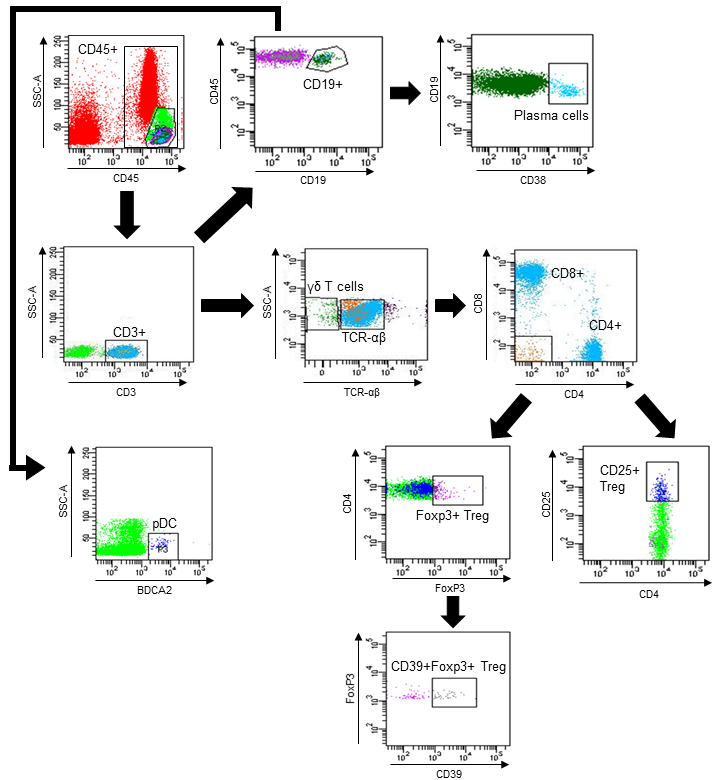

Supplement: sj-png-1-iji-10.1177_20587384211068667 – Supplemental Material for Patients with treated autoimmune hepatitis and persistent suppression of plasmacytoid dendritic cells: A different point of view [file sj-png-1-iji-10.1177_20587384211068667.png]
